# Effectiveness and safety of Shugan Jianpi (SGJP) formula in the treatment of nonalcoholic steatohepatitis (NASH): A protocol for systematic review and meta-analysis of randomized controlled trials

**DOI:** 10.1097/MD.0000000000028366

**Published:** 2021-12-23

**Authors:** Mingtai Chen, Yanghui Gu, Furong Huang, Guofu Zhong, Ling Men, Qiang Liu, Jienan Luan, Guangdong Tong

**Affiliations:** a Department of Cardiovascular Disease, Shenzhen Traditional Chinese Medicine Hospital, Shenzhen, China; b Department of Liver Disease, Shenzhen Traditional Chinese Medicine Hospital, Guangzhou University of Chinese Medicine, Shenzhen, China; c Intensive Care Unit, Shenzhen Traditional Chinese Medicine Hospital, Guangzhou University of Chinese Medicine, Shenzhen, China; d Nephrology Department, Shenzhen Traditional Chinese Medicine Hospital, Shenzhen, China.

**Keywords:** meta-analysis, nonalcoholic steatohepatitis, protocol, randomized trial, Shugan Jianpi

## Abstract

**Background:**

It is known that nonalcoholic steatohepatitis (NASH) has been more and more popular in clinical practice. Apart from lifestyle modification, pharmacological therapy treating NASH has still been limited and insufficient. A growing number of studies demonstrated that Shugan Jianpi (SGJP) formula, as a kind of Chinese herbal medicine prescription, could improve blood lipid indexes, liver function, and other clinical measures in NASH patients. Nevertheless, there still has been a lack of study to systematically assess the efficacy and safety of SGJP formula treating NASH. Therefore, it is necessary to conduct this systematic review and meta-analysis.

**Methods:**

A systematic literature search for articles up to December 2021 will be performed in following electronic databases: MEDLINE, Embase, the Cochrane Library, China National Knowledge Infrastructure, Chinese Scientific Journals Database Database, Chinese Biomedical Database, Chinese Biomedical Literature Service System, and Wanfang Database. Inclusion criteria are randomized controlled trials of SGJP formula applied on NASH patients. The primary outcome measures will be liver function, blood lipid indexes, ultrasound, or radiological imaging examination. The safety outcome measures will be adverse events and kidney function. RevMan 5.3 software will be used for data synthesis, sensitivity analysis, subgroup analysis, and risk of bias assessment. A funnel plot will be developed to evaluate reporting bias. Stata 12.0 will be used for meta-regression and Egger tests. We will use the Grading of Recommendations Assessment, Development and Evaluation system to assess the quality of evidence.

Discussion: This study will provide a high-quality synthesis of the efficacy and safety of SGJP for NASH patients.

**Ethics and dissemination:**

This systematic review does not require ethics approval and will be submitted to a peer-reviewed journal.

**Trial registration number:**

PROSPERO CRD42021259097.

## 1 Introduction

It is known that nonalcoholic steatohepatitis (NASH) has been a growing concerned issue accompanying with severe economic and health burden.^[1]^ The mechanism of the NASH progression is comprehensive, including lipid metabolism dysfunction, insulin resistance, oxidative stress, inflammation, fibrosis, etc.^[1–3]^ Apart from lifestyle modification and bariatric surgery, there has been still a lack of definite pharmacological treatment measures for NASH.^[4]^ More and more evidences from clinical studies have demonstrated that Chinese herbal medicine (CHM) is of benefit in the treatment of NASH in multiple aspects. Thus, CHM might provide a potential therapeutic strategy for treating NASH.^[5]^

CHM formulas have been applied in preventing and treating liver diseases for thousands of years. Based on the clinical manifestations of traditional Chinese medicine theory, NASH is classified into category of “Gan-Pi” and “Xie-tong.”^[6]^ The dominant symptoms of NASH are associated with qi stagnation and spleen deficiency. Thus, Shugan Jianpi (SGJP) prescription has been formulated to treat NASH according to the related symptoms above. SGJP formula mainly includes Herba Gynostemmatis Pentaphylli, Radix Curcumae, Rhizoma Atractylodis Macrocephalae, Poria, Rhizoma Alismatis, Herba Silybi, Semen Cassiae, Radix Salviae Miltiorrhizae, White Mustard Seed, and Fructus Crataegi.^[7]^ Although an increased number of clinical studies have assessed SGJP in the treatment of NASH patients, there has been a lack of systematic review to evaluate the efficacy and safety of SGJP in such patient population. In view of the shortcomings of previous studies and the insufficient evidence regarding the widespread application of SGJP formula, this systematic review aimed to summarize the efficacy and safety of SGJP formula in treating NASH patients.

## 2 Methods and analysis

### 2.1 Registration

The study protocol has been registered in the international prospective register of systematic review. The trial registration number of the international prospective register of systematic review is CRD42021259097. The procedure of this protocol will be conducted according to the preferred reporting item for systematic review and meta-analysis protocols guidelines.^[8]^

### 2.2 Eligibility criteria

#### 2.2.1 Type of study

##### 2.2.1.1 Inclusion

We will include all the randomized controlled trials that investigated the efficacy and safety of SGJP only or combined with conventional pharmacotherapy for the treatment of NASH.

##### 2.2.1.2 Exclusion

The studies will be excluded if it is not an randomized controlled trial (namely, observational cohort and case–control studies, case reports, experimental studies, and reviews).

#### 2.2.2 Participants

Inclusion: The study will include adult (18–85 years) NASH patients regardless of sex, ethnicity, education or economic status, and whether or not they were out- or in-patients. The diagnostic criteria for NASH will be as follows. The diagnostic criteria of NASH should be confirmed according to one of the past or current definitions: “Guidelines for NAFLD 2010” / “Guidelines for NAFLD 2018” / “AASLD Guidelines for NAFLD 2012” / “WGO Guidelines for NAFLD 2014.”

##### 2.2.2.1 Exclusion

Patients with severe respiratory disease, acute infectious disease, severe heart disease, severe liver disease, or tumors will be excluded.

### 2.3 Interventions

Inclusion: Eligible interventions will be those involving a combination of SGJP and conventional pharmacotherapy. The same conventional pharmacotherapy must be used in the control group.

#### 2.3.1 Exclusion

Trials that include other co-interventions such as another herbal formula, acupuncture, cupping, moxibustion, massage, yoga, qigong, Tai Chi, or aromatherapy will be excluded.

### 2.4 Outcome

#### 2.4.1 Inclusion

The primary outcome measures will include the following: steatosis grade evaluation (the improvement or change level of hepatic steaosis mainly assessed by ultrasound or other radiological examinations), blood lipid indexes (total cholesterol, triglyceride, low-density lipoprotein cholesterol, and high-density lipoprotein cholesterol), and liver function (alanine aminotransferase-glutamate pyruvic transaminase, aspartate aminotransferase-glutamate pyruvic transaminase, or gamma glutamyl transpeptidase). The secondary outcome measures will include the following: inflammatory indexes (tumor necrosis factor [TNF]-α, interleukin [IL]-6, or hypersensitive-c-reactive-protein [hs-CRP]), oxidative stress indexes (superoxide dismutase [SOD], malondialdehyde [MDA], or glutathione peroxidase [GSH-Px]), islet function (fasting blood glucose [FBG], fasting insulin [FINS], or insulin resistance index [HOMA-IR]), and traditional Chinese medicine syndrome scale. The safety outcomes will include the following: adverse events (such as digestive symptoms, headache, dizziness, skin rash, etc), kidney toxicity measured by serum markers.

#### 2.4.2 Exclusion

The outcome measures not requested in this study will be excluded.

### 2.5 Search strategy

The following electronic bibliographic databases will be searched from inception to December 2021: MEDLINE, Embase, the Cochrane Library, China National Knowledge Infrastructure, Chinese Scientific Journals Database Database, Chinese Biomedical Database, Chinese Biomedical Literature Service System, and Wanfang Database. A manual search of key journals and of the reference lists of reviews captured by the initial searches will also be performed. There will be no limits on the language of publication. Only clinical trials will be included and searched. The following sources will also be searched to identify clinical trials that are in progress or completed: Clinical Trials.gov and WHO clinical trials registry. Any additional relevant studies will also be retrieved from the reference lists of systematic reviews and included studies. If possible, we will map search terms to controlled vocabulary. In addition, the search strategy for selecting the fields of title, abstract, or keyword will differ depending on the characteristics of the databases. Search terms will be grouped into 3 blocks (see Table 1).

**Table 1:** Search items.

Search block	Search items
Participants	Non alcoholic Fatty Liver Disease OR NAFLD OR Nonalcoholic Fatty Liver Disease OR Fatty Liver, Nonalcoholic OR Fatty Livers, Nonalcoholic OR Liver, Nonalcoholic Fatty OR Livers, Nonalcoholic Fatty OR Nonalcoholic Fatty Liver OR Nonalcoholic Fatty Livers OR Nonalcoholic Steatohepatitis OR Nonalcoholic Steatohepatitides OR Steatohepatitides, Nonalcoholic OR Steatohepatitis, Nonalcoholic OR NASH
Intervention	Shugan Jianpi OR Shuganjianpi OR SGJP OR Jianpi Shugan OR Jianpishugan OR JPSG OR Drugs, Chinese Herbal OR Chinese Drugs, Plant OR Chinese Herbal Drugs OR Herbal Drugs, Chinese OR Plant Extracts, Chinese OR Chinese Plant Extracts OR Extracts, Chinese Plant OR Medicine, Chinese Traditional OR Traditional Chinese Medicine OR Chung I Hsueh OR Hsueh, Chung I OR Traditional Medicine, Chinese OR Zhong Yi Xue OR Chinese Traditional Medicine OR Chinese Medicine, Traditional
Study design	Randomized controlled trial OR controlled clinical trial OR randomized OR placebo OR drug therapy OR randomly OR trial OR groups

### 2.6 Study selection and data extraction

**Figure 1:**
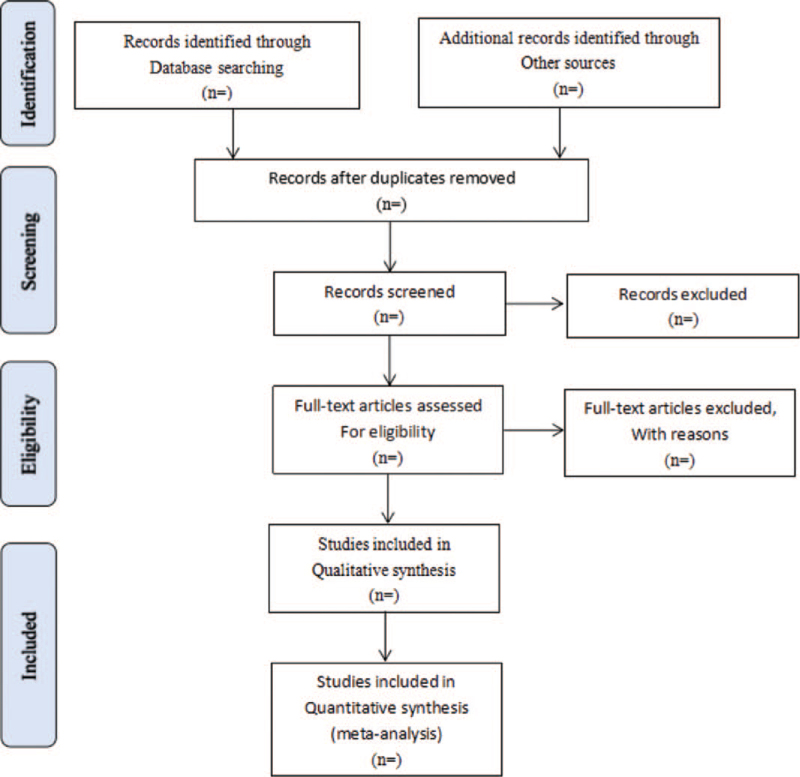
Flow diagram of study selection process.

Literature retrieved citations will be managed by EndNote X7 software (Clarivate Analytics, London, United Kingdom). Two authors (CM and HF) will independently screen the titles and abstracts of all the studies retrieved in the above electronic databases to identify potentially eligible studies. Articles that are duplicated or have not met the eligibility criteria, interventions, and outcomes in this study will be excluded. After filtering the final eligible articles, the data from the included articles will be extracted independently by 2 authors (ZG and ML). Disagreements will be resolved by discussion or arbitrated by a third author if needed. The following categories of data will be extracted: first author, publication year, diagnose information, age, sex, trial characteristics, interventions and controls, participants, study methodology, outcomes, and adverse events (see Fig. 1).

### 2.7 Risk of bias assessment

The methodological quality of the eligible studies will be evaluated according to the Cochrane Collaboration's tool for assessing risk of bias. The assessment details include: sequence generation, allocation concealment, blinding of participants and personnel, blinding of outcome assessors, incomplete outcome data, selective reporting, and other sources of bias. Each domain will be assessed as “low risk,” “high risk,” or “unclear risk” according to the description details of eligible studies.

### 2.8 Data synthesis and statistical analysis

Statistical analyses will be conducted with RevMan 5.3 software provided by Cochrane Collaboration (London, United Kingdom). The overall effect sizes will be determined as the mean difference for continuous outcomes, the odds ratio for dichotomous outcomes with their 95% credible intervals. The *Q* and *I*^2^ test statistics will be calculated to determine the amount of heterogeneity. For the *Q* statistic, *P* < .05 will be considered to indicate significant differences. For the *I*^2^ statistic, *I*^2^ < 25% indicates no significant heterogeneity, *I*^2^ = 25–50% is considered moderate heterogeneity and *I*^2^ > 50% indicates strong heterogeneity. We will use fixed effects models if there is no heterogeneity among studies, and random effects models if there is heterogeneity.

### 2.9 Sensitivity analysis, subgroup analysis, and meta-regression

If the heterogeneity or inconsistency among the studies is detected, a sensitivity analysis or subgroup analysis or meta-regression (conducted by Stata 12.0) analysis will be performed. Subgroup analysis will be conducted to explore potential sources of heterogeneity according to the characteristics of studies, including sample size, severity of NASH, dose of SGJP, treatment duration, and other relevant parameters. If data extraction is insufficient, we will create a qualitative synthesis.

### 2.10 Publication bias

A funnel plot will be developed to evaluate reporting bias of the included studies. We will use Egger tests (conducted by Stata 12.0) to assess funnel plot symmetry and will interpret values of *P* < .1 as statistically significant.

### 2.11 Quality of evidence

We will also assess the quality of evidence for the main outcomes with the Grading of Recommendations Assessment, Development and Evaluation approach. Five items will be investigated, including limitations in study design, inconsistency, inaccuracies, indirectness, and publication bias.

### 2.12 Patient and public involvement

The patients and/or public will not be involved because this study uses secondary sources for analysis.

## 3 Discussion

NASH has been considered as one of the main causes of chronic liver disease, cirrhosis and carcinoma.^[1,2]^ Even the profound and lasting hazard of NASH has been aware of by public, the solution to treat this disease has still be limited.^[9,10]^ More and more studies demonstrated that SGJP prescription could treat NASH patients in multiple aspects.^[7,11,12]^ In spite of this, there has been no comprehensive evaluation of the clinical evidence regarding SGJP as intervention for NASH patients in evidence-based medicine. Accordingly, we aim to operate this systematic review to evaluate the efficacy and safety of SGJP in the treatment of NASH. However, there still might be several drawbacks for this is a retrospective systematic review. For instance, unpublished studies might not be identified introducing some bias. In addition, gray literature might be difficult to retrieve causing selection bias. Furthermore, several secondary outcome measures might not be completely reported. Nevertheless, this study is expected to propose clinical recommendations for NASH patients in clinical practice and provide more objective and reliable evidence supporting use of the SGJP.

## Author contributions

Tong Guangdong and Chen Mingtai conceived the study and drafted the protocol. Gu Yanghui, Luan jienan, and Liu Qiang revised it. Chen Mingtai, Huang Furong, Men Ling, and Zhong Guofu developed the search strategies, will conduct data collection, and analyze the data independently. All authors will approve the final manuscript.

**Conceptualization:** Mingtai Chen, Yanghui Gu, Ling Men, Jienan Luan, Qiang Liu, Guangdong Tong.

**Data curation:** Mingtai Chen.

**Formal analysis:** Mingtai Chen, Guofu Zhong.

**Funding acquisition:** Yanghui Gu, Jienan Luan, Guangdong Tong.

**Investigation:** Furong Huang.

**Methodology:** Yanghui Gu, Furong Huang, Guofu Zhong.

**Project administration:** Yanghui Gu, Ling Men, Qiang Liu.

**Resources:** Furong Huang, Guofu Zhong.

**Software:** Furong Huang, Guofu Zhong.

**Supervision:** Ling Men, Jienan Luan, Qiang Liu, Guangdong Tong.

**Validation:** Furong Huang.

**Visualization:** Guofu Zhong, Ling Men.

**Writing – original draft:** Mingtai Chen, Yanghui Gu.

**Writing – review & editing:** Mingtai Chen, Jienan Luan, Qiang Liu, Guangdong Tong.
